# Michigan

**Published:** 1896-08

**Authors:** 


*Michigan.* Professor E. A. A. Grange has discovered tuberculosis among the State College herd.

